# Achieving high uptake of human papillomavirus vaccination in Malaysia through school-based vaccination programme

**DOI:** 10.1186/s12889-018-6316-6

**Published:** 2018-12-22

**Authors:** Nor Asiah Muhamad, Saidatul Norbaya Buang, Safurah Jaafar, Rohani Jais, Phaik Sim Tan, Normi Mustapha, Noor Aliza Lodz, Tahir Aris, Lokman Hakim Sulaiman, Shahnaz Murad

**Affiliations:** 1 Institute for Public Health, Ministry of Health, Kuala Lumpur, Malaysia; 2 Family Health Development Division, Ministry of Health, Kuala Lumpur, Malaysia; 3 Department of Community Medicine, International Medical University, Kuala Lumpur, Malaysia; 4 Faculty of Science and Technology, Open University Malaysia, Kuala Lumpur, Malaysia; 5 Disease Control Division, Ministry of Health, Kuala Lumpur, Malaysia; 6 Office of Deputy Director General of Health (Public Health), Ministry of Health, Putrajaya, Malaysia; 7 Office of Deputy Director General of Health (Research and Technical Support), Ministry of Health, Putrajaya, Malaysia

**Keywords:** Cervical cancer, Immunisation, HPV, School based, Malaysia

## Abstract

**Background:**

In 2006, 4 years of planning was started by the Ministry of Health, Malaysia (MOH), to implement the HPV (human papillomavirus) vaccination programme. An inter-agency and multi-sectoral collaborations were developed for Malaysia’s HPV school-based immunisation programme. It was approved for nationwide school base implementation for 13-year-old girls or first year secondary students in 2010. This paper examines how the various strategies used in the implementation over the last 7 years (2010–2016) that unique to Malaysia were successful in achieving optimal coverage of the target population.

**Methods:**

Free vaccination was offered to school girls in secondary school (year seven) in Malaysia, which is usually at the age of 13 in the index year. All recipients of the HPV vaccine were identified through school enrolments obtained from education departments from each district in Malaysia. A total of 242,638 girls aged between 12 to 13 years studying in year seven were approached during the launch of the program in 2010. Approximately 230,000 girls in secondary schools were offered HPV vaccine per year by 646 school health teams throughout the country from 2010 to 2016.

**Results:**

Parental consent for their daughters to receive HPV vaccination at school was very high at 96–98% per year of the programme. Of those who provided consent, over 99% received the first dose each year and 98–99% completed the course per year. Estimated population coverage for the full vaccine course, considering also those not in school, is estimated at 83 to 91% per year. Rates of adverse events reports following HPV vaccination were low at around 2 per 100,000 and the majority was injection site reactions.

**Conclusion:**

A multisectoral and integrated collaborative structure and process ensured that the Malaysia school-based HPV immunisation programme was successful and sustained through the programme design, planning, implementation and monitoring and evaluation. This is a critical factor contributing to the success and sustainability of the school-based HPV immunisation programme with very high coverage.

## Background

HPV is the commonest sexually transmitted infection (STI) [1, 2]. HPV infection is associated with various health problems such as genital warts, cancers of male and female genitalia and of the oropharynx [2–4]. These double-stranded deoxyribonucleic acid (DNA) viruses are the main causative agents in cervical intraepithelial neoplasia and cancer [5]. HPV genotypes 16 and 18 have been established to cause 70% of cervical malignancies in women [2, 6].

### Cervical cancers incidence

Cervical cancer is the second most frequent cancer in the low-income countries and fourth in the world [7].It is the most common malignant cancer of the female reproductive organs [8, 9]. Globally, the incidence of cervical cancer is estimated to be at 500,000 cases with 300,000 deaths yearly. About 80–85% of these cases occur in developing countries [9–11]. In Malaysia, cervical cancer is the second most frequently occurring cancer (after breast cancer) amongst women aged 15–44 years [12]. Between 2007 and 2011, a total of 4352 new cases of cervical cancer were reported by the National Cancer Registry. Of these cases, 34.5% occurred among women aged 50–59 years and 64.4% were detected at stage 1 and 2 [13, 14].

### Screening and prevention for cervical cancer

Various strategies for prevention have been developed including vaccination, cervical cytology (Pap testing), and HPV DNA based screening, for early detection and effective treatment. HPV vaccines covering HPV16 and 18 can prevent up to 70% of cervical cancer cases without screening [15, 16]. Early screening and detection will significantly increase the chance of survival as the cellular changes are detected earlier and the treatment can commence immediately [17]. Since HPV vaccination was introduced in 2006, more than 71 countries have adopted HPV vaccination as a primary strategy for the prevention of cervical cancer [18]. The HPV Immunisation Programme in Malaysia was launched in August 2010 and added to the list of the National Immunisation Programme, which provides selected vaccines free of charge to all residents as a public health service [19]. The population of Malaysia in 2016 was approximately 32 million comprising predominantly Malay, Chinese, Indian and others, including indigenous groups in Peninsular Malaysia and the native people of East Malaysia, as compared to 32.2 million in 2017, with an annual population growth rate of 1.1% with most people residing in suburban and rural areas [20].A study done by Cheong et al. [21] and Norrafizah et al. [22] showed that people in rural areas and with low socio-economic status have poorer health literacy.

### Screening practices in Malaysia

Prior to 2010, cytology-based screening was the main preventive measure for cervical cancer in Malaysia. The Papanicolaou smear (Pap smear) screening services initially aimed at post-partum mothers in family planning programs. In 1995, Pap smear screening was expanded to women aged between 20 to 65 years [23]. Despite the availability and promotion campaign on screening services nationwide, the uptake rates were only 26% in 1996 and 43.7% in 2006 [24]. Lack of knowledge about the disease, inadequate health literacy, low perception of cancer risk, behaviour and attitude towards the preventive programme and lack of systematic, population based, active recruitment approaches to women were the factors that contributed to the low percentage of women seeking and receiving the service [21, 22, 25]. Therefore, an HPV vaccination programme was developed for school girls in Malaysia as part of its cancer prevention strategy.

The HPV vaccines became available in 2006 [26]. Since then, efforts were initiated to introduce the HPV vaccination in Malaysia. The vaccines (Gardasil and Cervarix) were registered for private market use in 2006 and 2010 respectively for women aged 9 to 40 years [27, 28]. The introduction of HPV vaccination was projected to prevent 89% of cervical cancer caused by HPV 16 and HPV 18 [29] and save substantial annual costs for HPV related treatments [30, 31].

The Ministry of Health (MOH), through the school-based services, has introduced the HPV vaccination program for school girls at age 13. The school health service package provides school-based services from preschool until age 15 as shown in Table 1. The HPV vaccination program is projected to reduce occurrence of the cervical cancer incidence associated with HPV16 & 18 among immunised girls in next 20 years. This paper aims to describe the new policy and model for delivery of HPV vaccination, and the initial experiences and results from pilot implementation from the Malaysian school base HPV program vaccination and factors contributing to its successful implementation over the last 6 years.

**Table 1:** Package of school health services in MOH

Grade & Age	Service Package
Pre school	Growth and development Assesment
Standard 1 (age 7)	• Health Education, Health Appraisal, Vision screening, BMI Monitoring Immunization Measles Rubella (MR) and Diphtheria Tetanus (DT) Booster dose)
Standard 3 (age 7)	Learning Disability Assessment and Confirmation
Standard 6 (age 11)	• Health Education, Health Appraisal; Vision screening; BMI Monitoring and • Scoliosis screening
Form 1 (girls age 13)	• HPV Immunization
Form 3 (age 15)	• Health Education, Health Appraisal, Vision screening, BMI Monitoring and • Color Defect Screening; ATT Booster Immunization
Form 4 (age 16)	• Thalassaemia Screening

Source: Family Health Department, Ministry of Health Malaysia 2018

### Vaccination schedule

Intramuscular injection of 3 doses of quadrivalent HPV vaccine delivered on a standard dosing schedule (at 0, 2, and 6 months).From 2010 to 2014, each recipient received 3 doses of HPV vaccine at the interval of 0, 1 and 6 months. However, in 2015 following the guideline from the World Health Organization (WHO), two doses of HPV vaccine were introduced under the new policy where by these doses of vaccine were given at 0- and 6-monthsinterval [32, 33]. This school base grade cohort approach was preferred, as it clearly defined the target population and allowed precise estimates of vaccine doses and consumables required to be procured.

## Methods

### Description of process on HPV vaccination program

#### Integration of HPV vaccination into National Immunisation and school health services

High rates of school enrolment for 13-year olds (96.0%) and retention of female students in secondary schools have made it possible for the HPV vaccination to be integrated into the School Health Service Program and ensures equal access to the HPV vaccine between urban and rural area [20, 24].

#### MOH Organisational infrastructure

At the onset of the launch, 450 school’s health teams were readily available to deliver the services throughout the country for both public and private schools. To ensure the 3 doses of vaccination were able to be delivered within the set time frame, the nurses from the various MOH health clinics were mobilised to support the school health teams.

#### Reporting

The progress of implementation on the level of consent and coverage of immunization were reported to National Policy and Practices on Immunisation committee regularly. The year seven female students’ enrolment data were obtained from District Education departments at the end of the year and later verified with schools’ enrolment lists.

#### Program monitoring

The most crucial factor that determines supporting high vaccination coverage was program monitoring. The National, State and districts HPV Operation Rooms were set-up to closely monitor the implementation at all implementation levels. The operation room was spearheaded by the Director of the Family Health Development Division, with support from various technical officers responsible for technical documentation of the progress, adverse reactions, public enquiries, ICT requirements and daily operations. All public enquiries on the HPV immunization were responded to by a Public Health Physician and paramedics that were put on-call on a rotational basis to answer the hotlines from the public as well as any queries from implementers at school or health clinics.

#### Program approach

The National School based HPV Immunisation Program was implemented in 2958 public and private secondary schools registered under the Ministry of Education, Malaysia (MOE). A scheduled school visit by the school health team was carried out to ensure HPV vaccination spaced at month 0, 1 and 6 were completed within the same year before school end in November.

#### Consent approach

Written parental consent was obtained prior to vaccination. The consent forms were distributed to parents through school teachers prior to scheduled vaccination visits. The parents returned the consent form to the school team. Whenever the children were refused immunization by their parents, the school health teams would call the parents to determine reasons for refusal and made are mark on the student registration form.

#### Ethical issues

Ethical issues surrounding HPV vaccination and parental rights to adequate information was not compromised in the process of obtaining parental written consent. Parents were provided with HPV vaccine information through print and electronic media to enable those making independent informed choices.

Compliance to religious requirement was fundamental to HPV vaccine uptake amongst the Muslim population in Malaysia. MOH worked with Malaysian religious authority in having an Islamic ruling (fatwa) on HPV vaccination. The ruling stipulates “Immunising women with HPV vaccine that has been determined to have no element of doubt in its content and will not harm the recipient, is permissible for the prevention of cervical cancer” [34, 35].

#### Managing risk communication

Introduction of HPV vaccine particularly involves supporting the acceptability of the vaccines to adolescent girls who may have little or no knowledge about cervical cancer and parents that may be suspicious of the vaccines, will give particularly if exposed to negative media attention and thus programs must proactively address communication requirements [36, 37]. Through an effective health communication programs, HPV vaccination was successfully introduced and found broad-based support from policy decision-makers, healthcare professionals, and the general public as well as by the target population [38].

#### Social mobilisation and advocacy

**Fig. 1:**
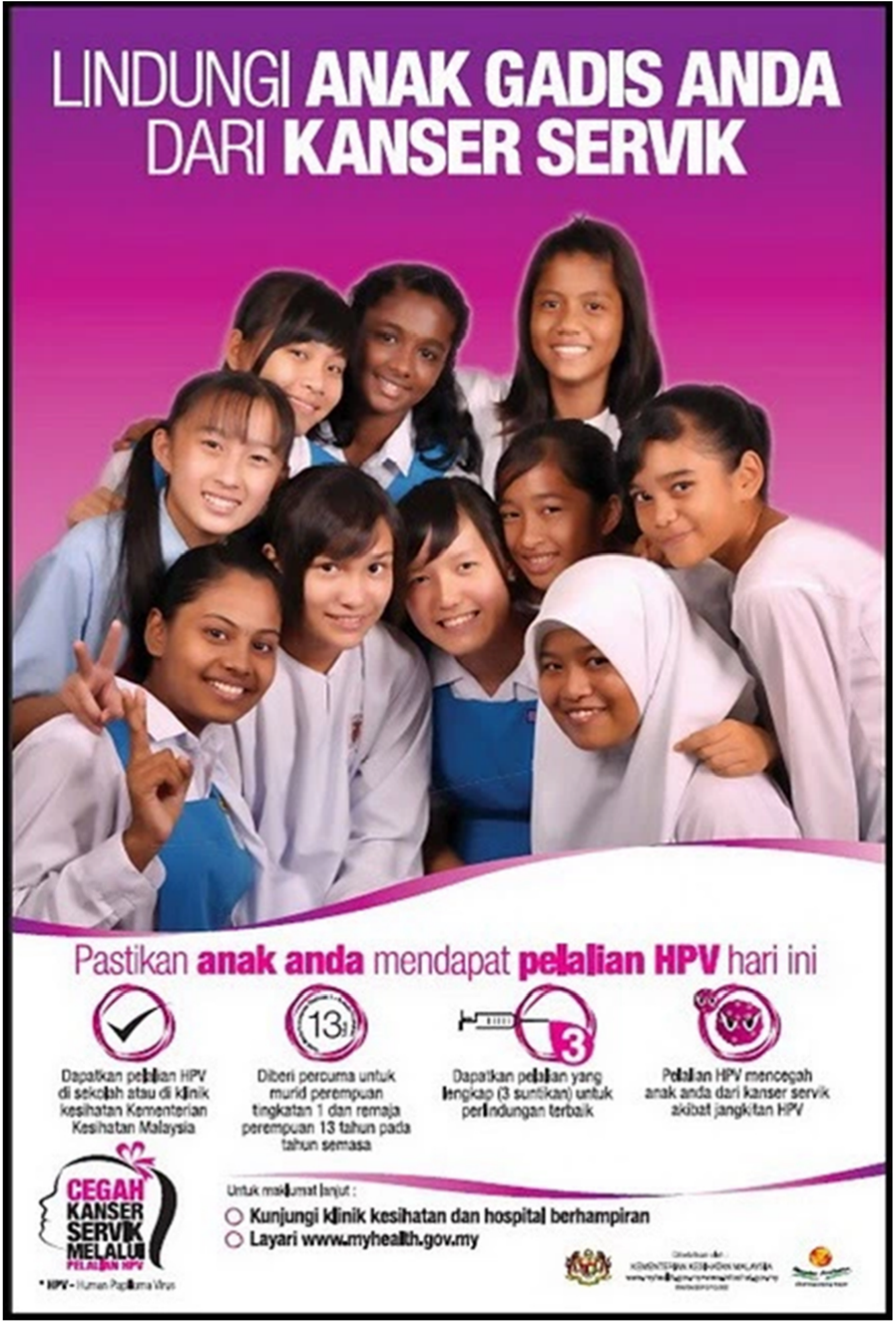
Tagline (Translation “Lindungi Anak Gadis Anda dari Kanser Serviks”: Protect Your Daughter from Cervical Cancer) (Translation “Pastikan anak anda mendapat pelalian HPV hari ini”: Ensure your daughter receives HPV vaccination today) (Translation “Dapatkan pelalian HPV di sekolah atau di klinik kesihatan Kementerian Kesihatan Malaysia”: Get the HPV vaccination in school or government health clinic, Ministry of Health Malaysia) (Translation “Diberi percuma untuk murid perempuan tingkatan 1 dan remaja perempuan 13 tahun pada tahun semasa”: Given free of charge to form 1 school girl and adolescent girl aged 13 in the index year) (Translation “Dapatkan pelalian yang lengkap (3 suntikan) untuk perlindungan terbaik”: Get complete HPV vaccination (3 injections) for best protection)(Translation “Pelalian HPV mencegah anak anda dari kanser servik akibat jangkitan HPV”: HPV vaccination prevent your daughter from cervical cancer due to HPV infection) (Translation “Untuk maklumat lanjut: Ο Kunjungi klinik kesihatan dan hospital berhampiran atau Layari www.myhealth.gov.my”: For further information: Visit nearest clinic and hospital or surf www.myhealth.gov.my)

Electronic, printed and social media were the main communication channels employed in creating public awareness towards accepting HPV vaccination [39–41]. Major telecommunication stations, newspapers and magazines were engaged to air the HPV messages 5 months prior to starting the programme (Fig. 1). Multiple campaigns regarding the vaccines and its availability were constantly featured at prime time on main communication channels and on the bill board. Traditional and social media outlets were very active during the first 2 years of the launch. Information such as the programme website link was provided in every pamphlet and poster and also in electronic media. A specific tagline and a logo were created by Health Education Division, Department of Public Health, MOH for the female students to associate themselves with the HPV vaccination (Fig. 1).Negative feedback and reporting on HPV vaccination abroad and locally were closely monitored and addressed accordingly to alleviate fear among the public and the target population.

On the day of the vaccination, the school health nurses delivered a brief introduction on the HPV infection, cervical cancer, and HPV vaccine to the recipients. After the introduction, an assessment was carried out on health status to determine presence of illness or any contraindications for vaccination which require immunisation to be deferred to later dates. A guideline was developed by Family Health Development Division, MOH to carry out the mass vaccination [42]. The HPV injections were given by the community nurses. Relevant individual information on the vaccination were captured and documented in HPV vaccination registration forms and individual vaccination cards.

After the vaccination, recipients were observed for 20 min for any immediate adverse reactions. They were provided with an Adverse Event Following Immunisation (AEFI) monitoring form and vaccination card before returning to their classes. All recipients were advised to seek medical help if they experienced any suspected adverse reactions. The AEFI monitoring forms were collected from students at school either on the following visits or at any contact with the health providers in accordance to guidelines [24].

## Results

### Delivery of HPV vaccination

#### Eligible criteria for HPV vaccination

Free vaccination was offered to eligible recipients (year seven) in Malaysia which was usually at the age of 13 in the index year. Only female year seven students are eligible to receive vaccination irrespective of their actual age. All recipients of the HPV vaccine were identified through school enrolment data obtained from education departments from each district in Malaysia. A total of 242,638recipients aged between 12 to 13 years studying in year seven were approached during the launch of the program in 2010. Approximately, 230,000 recipients were offered HPV vaccine annually by 646 school health teams throughout the country from 2010 to 2016.

#### Monitoring indicators

**Fig. 2:**
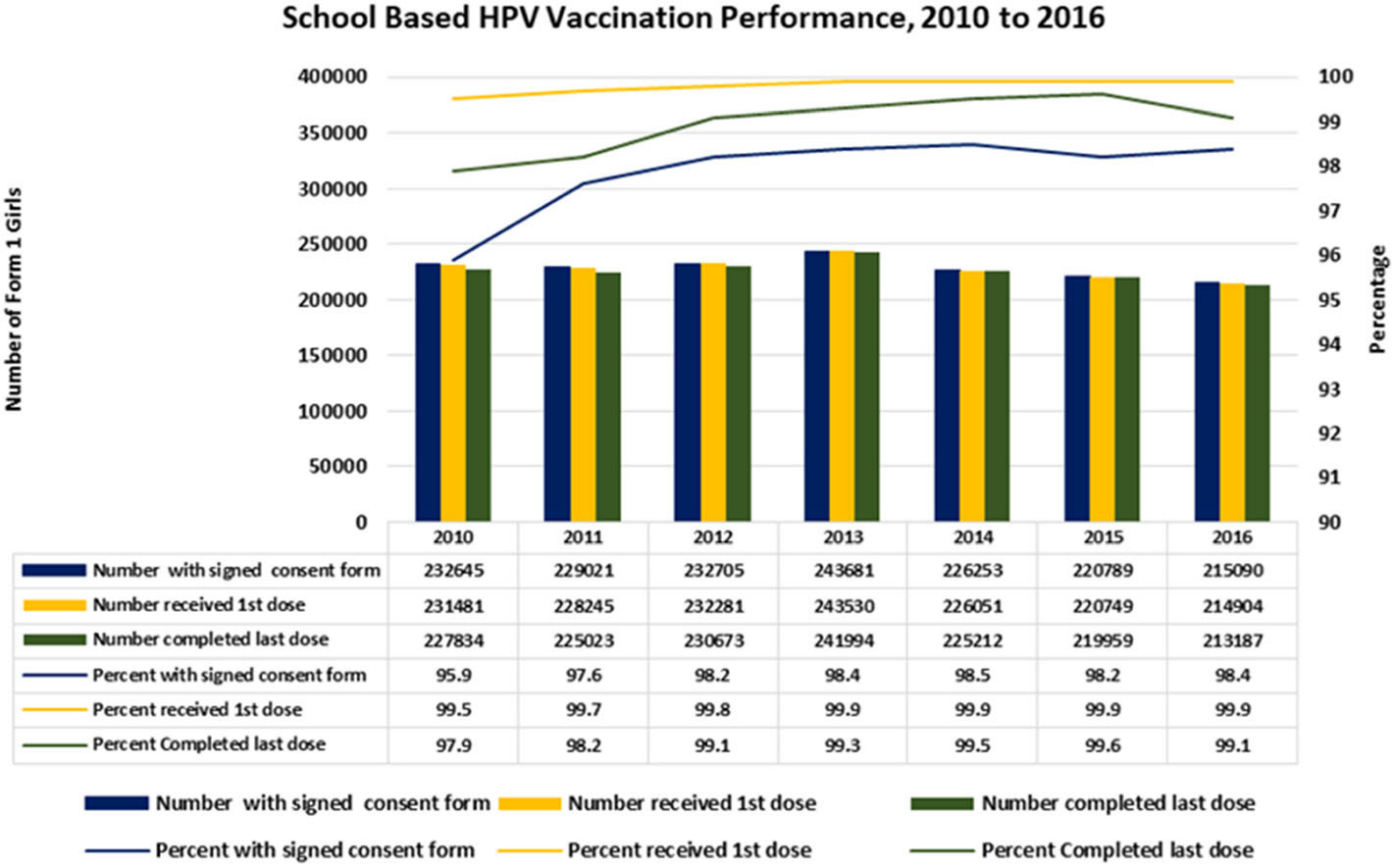
Percent of girls with parental consent who had first and third vaccination dose, 2010–2016

Parental consent, coverage by doses and vaccination completion were closely monitored as part of vaccination delivery indicators. Parental consent reflects the level of acceptance of the vaccine introduced. It was measured by the total number of parents consented for their child to be vaccinated over total number of year seven female student’s enrolment. A total of 232,645 from 242,638 (95.9%) parents consented to the immunisation in 2010. Subsequently, in 2011 a total of 229,021 from 234,668 (97.6%) and in 2012 a total 232,705 from 237, 017 (98.2%) were obtained from the parents. Throughout 2013, a total of 243,681 from 247,549 (98.4%) parents gave consent for immunisation. In 2014, 2015 and 2016, a total of 226,253 from 229,739 (98.5%), 220,789 from 224,761 (98.2%) and 215,090 from 218,590 (98.4%) parents consented to their child to be immunised respectively. Reasons for non-consent included unsure of vaccine safety, child has been immunised in private clinic and unable to retrieve consent forms from students. Figure 2 showed the percent of girls with parental consent who completed the vaccination.

#### Vaccination coverage

**Fig. 3:**
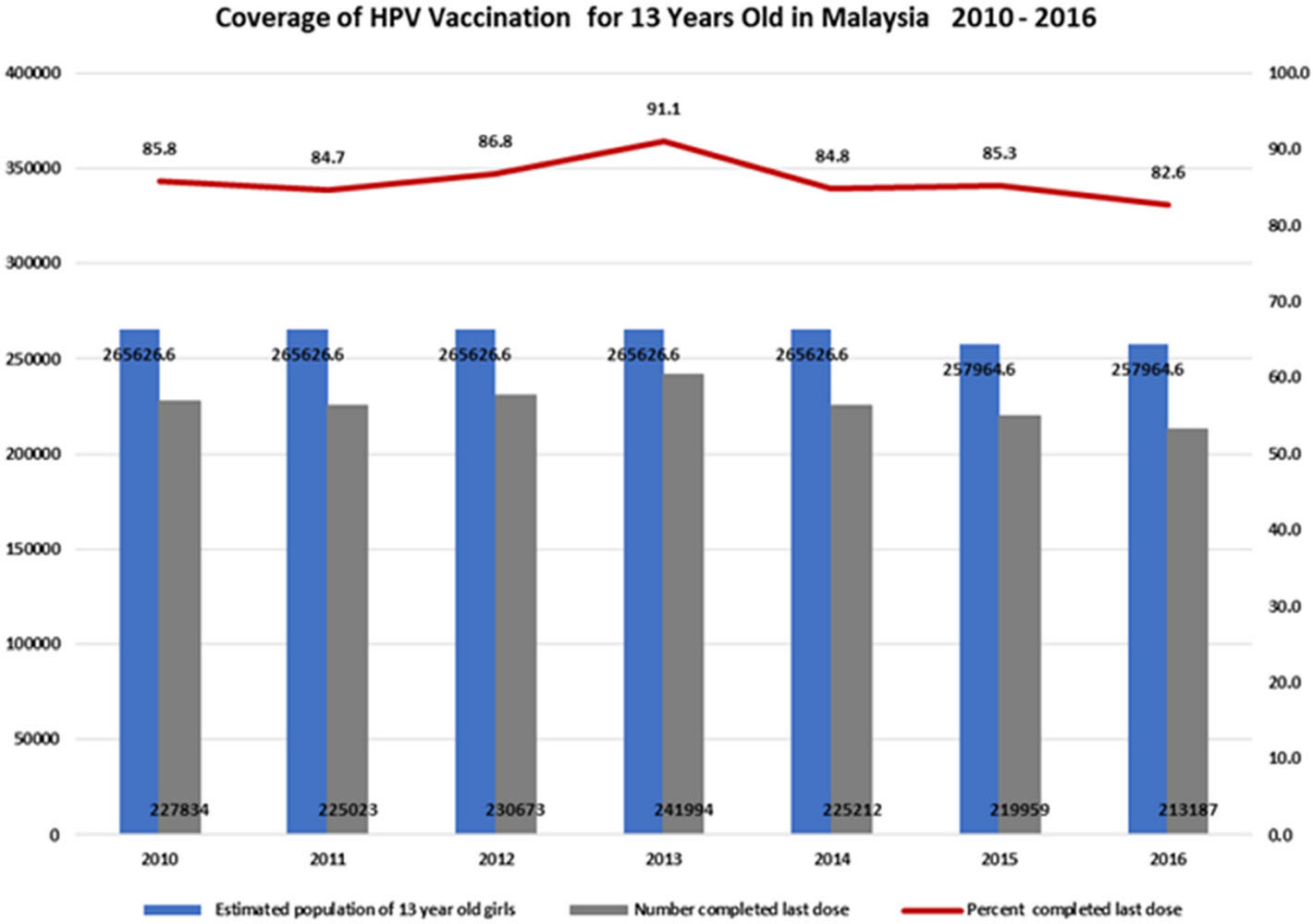
Percent of 13 year old girls vaccinated, 2010–2016 *Source for all figures: Family Health Department, Ministry of Health Malaysia 2018

Vaccination coverage provides information on vaccination uptake in the total population, including those girls not in school and therefore not offered vaccination. It was measured by total number of recipients who received a complete immunisation course over estimated total number of female students (Fig. 3).

#### Vaccination completion

Completed vaccination refers to recipients completing 3 doses of HPV vaccination or 2 doses in 2015. It was observed that 93.35% students with parental consent completed their 3 doses of vaccination in 2010. It improved to 98.3% in 2011 and increased to 99.3, 99.4 and 99.6% in 2012, 2013 and 2014, 99.6% from 2015 and 99.1% from 2016 cohort who received 2 doses.

#### Dose 1 completion

First dose completion for 2010 was 99.5% for recipients with parental consent. The completion improved to 99.7, 99.8 and 99.9% from 2012, 2013 and 2015 respectively but declined to 83% in 2016. Main reason for non-immunised among the consented were fear, absenteeism, claimed to have been immunised before and due to vaccine being charged to the students attending private sector schools in 2016.

#### Dose 2 completion

The completion rate for the second dose was slightly lower than the first dose. The completion of second dose was 98.9% in 2010 and increased above 99% in 2011. The main reason stated by students for not continuing their vaccination course was the experience of side effects during first dose.

#### Dose 3 completion

Third dose completion rates showed a further drop of on average 0.5 to 1%. The highest drop for the third dose was in 2010 from 98.9 to 97.9% while the lowest drop was 0.3% in 2014. Third dose completion was not applicable in 2015 due to the two doses schedule. Reasons for not completing were similar to the reasons stated for the second dose.

#### Vaccine safety

Active adverse event following immunisation (AEFI) monitoring was introduced to create students and parental awareness on vaccine and vaccination safety. Any adverse event would be reported for every dose of immunisation. Active reporting was encouraged by providing an AEFI form to all recipients. Reports of recipients experiencing AEFI were collected and monitored by the Malaysian Adverse Drug Reactions Advisory Committee (MADRAC).

During the first year of implementation, the MOH managed to provide HPV vaccination services to almost 96% (231,869) of eligible recipients in all public, private and international secondary schools. The absence of mothers during vaccination in school did not reduce the HPV vaccination uptake among girls in Malaysia [19, 43]. Throughout the 5-year period, the MOH received AEFI reports at a rate of about 2 per 100,000 doses administered (7872 AEFI reports out of 3.9 million HPV vaccination doses) as shown in Table 2. On average, two symptoms were notified in every report received. The main symptoms reported were itching, bruising or soreness at the administration site at 12184 (72.3%) followed by headaches and giddiness at 2166 (12.9%) and gastrointestinal disorder at 1461 (8.7%).

**Table 2:** Notifications of Adverse events following immunisation (AEFI)

	2010	2011	2012	2013	2014	2015	2016
Total dose of vaccine delivered	689,490	679,924	692,530	728,604	677,131	440,754	428,091
No. of AEFI reported	412	3033	1831	1606	908	1095	656
Percentage of AEFI reported	0.06	0.45	0.26	0.22	0.13	0.25	0.15

Source: National Pharmaceutical Regulatory Agency, Ministry of Health. 2018

## Discussion

The World Health Organization recommends a school-based approach for effectively delivering HPV vaccine in countries that have a fairly high enrolment of girls in schools [44]. The Malaysian experience demonstrates one model and some of the essential elements that contributed to achieving success with such an approach. School-based programmes require a stable commitment of and strong collaboration between health and education authorities.

### Political will and commitment

A sustainable national HPV vaccination programme is resource intensiveand requires a strong policyto ensure continuous financial support [45–47]. Advocacy plays a major role in shifting the political stakeholders’ views towards the importance of cervical cancer prevention programme to reduce the incidence of cervical cancer through prophylactic HPV vaccine [46]. Advocacy efforts produced fruitful results and was reflected in 2007 which was done by sharing education information through multimedia coverage and cost effectiveness of HPV vaccine against cervical cancer [29, 48].

### Smart partnership with relevant stakeholders

Partnerships across stakeholders are necessary for the development of relevant and effective strategies in addressing issues for maximum efficiency and positive impact of national HPV vaccination implementation [49, 50]. The existing relationship with the Ministry of Education (MOE) was an enabling factor in facilitation of voluntary parental consent through schools. Collaboration with MOE was strengthened at national, state, district and operational levels. Active monitoring by the personnel within MOE to all school principals to participate in the HPV vaccination program was done to enable the program to be conducted in systematic way and according to schedule. Interactive discussion between school administrative and school health teams was carried out regularly to ensure successful school immunization programme [51]. Regular discussions were held with relevant government agencies, professional bodies and non-government agencies to gain their continuous support and commitment.

While anticipating potential media threats, MOH utilized multipronged strategies to communicate the messages across the public. MOH had successfully engaged various subgroups to talk and discuss regarding the introduction of HPV immunization through print, electronic and social media (face book and twitter) and telephone hotline. Other activities carried out were rumours surveillance, public forums, health talks, print and electronic media campaigns. The messages were tailored to local cultural context, religion and information needs of parents, target population and general public and to alleviate fear and misconception about vaccine being new, and reinforce vaccine safety [52, 53].

## Conclusion

School-based programmes require the stable commitment of and strong collaboration between health and education authorities. Two essential elements for such collaboration are policies and mechanisms. National policies and the corresponding managerial and operational mechanisms are the key elements for the school health programme to be successful. The approach used in Malaysian school-based immunisation program is recommended as it was able to deliver HPV vaccination with good coverage. The success of the implementation in Malaysia was due to careful program planning at national, states and district levels. Involvement of various stakeholders and gatekeepers facilitated the public acceptance of a new and potentially controversial vaccine. Highlighting the relationship of the vaccination and cervical cancer in the context of the Malaysian culture and religious belief further enhanced the uptake of vaccination in school. Periodic mass media campaigns created a positive supportive environment in changing beliefs and practices towards the new vaccine. Availability of health personnel to respond to public concerns further increased public confidence with the MOH new policy. Political will and a high-level commitment ensure the sustainability of the School-based HPV Vaccination Programme in Malaysia.

## Abbreviations

AEFI: Adverse effects following immunization
DNA: Deoxyribonucleic acid
HPV: Human papilloma virus
MADRAC: Malaysian adverse drug reaction advisory committee
MOE: Ministry of education
MOH: Ministry of health
STI: Sexual transmitted illness
WHO: World Health Organization
